# The Genetic and Molecular Mechanisms of Congenital Hyperinsulinism

**DOI:** 10.3389/fendo.2019.00111

**Published:** 2019-02-26

**Authors:** Sonya Galcheva, Hüseyin Demirbilek, Sara Al-Khawaga, Khalid Hussain

**Affiliations:** ^1^Department of Paediatrics, University Hospital St. Marina, Varna Medical University, Varna, Bulgaria; ^2^Department of Paediatric Endocrinology, Hacettepe University Faculty of Medicine, Ankara, Turkey; ^3^Division of Endocrinology, Department of Paediatric Medicine, Sidra Medicine, Doha, Qatar

**Keywords:** hyperinsulinism, hypoglycaemia, molecular mechanisms, genetics, mutation

## Abstract

Congenital hyperinsulinism (CHI) is a heterogenous and complex disorder in which the unregulated insulin secretion from pancreatic beta-cells leads to hyperinsulinaemic hypoglycaemia. The severity of hypoglycaemia varies depending on the underlying molecular mechanism and genetic defects. The genetic and molecular causes of CHI include defects in pivotal pathways regulating the secretion of insulin from the beta-cell. Broadly these genetic defects leading to unregulated insulin secretion can be grouped into four main categories. The first group consists of defects in the pancreatic K_ATP_ channel genes (*ABCC8* and *KCNJ11*). The second and third categories of conditions are enzymatic defects (such as GDH, GCK, HADH) and defects in transcription factors (for example HNF1α, HNF4α) leading to changes in nutrient flux into metabolic pathways which converge on insulin secretion. Lastly, a large number of genetic syndromes are now linked to hyperinsulinaemic hypoglycaemia. As the molecular and genetic basis of CHI has expanded over the last few years, this review aims to provide an up-to-date knowledge on the genetic causes of CHI.

## Introduction

Congenital hyperinsulinism (CHI) is a heterogeneous and complex biochemical disorder which is characterized by the dysregulated release of insulin from pancreatic β-cell (1). In normal physiological state, the secretion of insulin is tightly coupled to glucose metabolism within the β-cell so that the insulin release is regulated to keep the plasma glucose concentration around 3.5–5.5 mmol/L. However, in CHI the secretion of insulin becomes unrelated to glucose metabolism, so that there is inappropriate insulin release for the plasma glucose level (2).

The genetic and molecular cause of CHI includes defects in key genes regulating insulin secretion from the pancreatic β-cell. Molecular defects in previously described genes (*ABCC8, KCNJ11, GLUD1, GCK, HADH, SLC16A1, UCP2, HNF4A, HNF1A, HK1, PGM1*, and *PMM2)* have been reported (3). However, recent studies have linked the role of other genes (*CACNA1D, FOXA2*) to hyperinsulinaemic hypoglycaemia (HH) but in some of these cases the underlying molecular mechanisms are still not fully elucidated (Table 1). Understanding the molecular mechanisms of CHI due to these genetic abnormalities has provided unique insight into the normal physiological mechanisms which regulate the insulin release.

**Table 1:** Monogenic causes of CHI.

**Gene**	**Protein**	**Function**	**Chromosome**	**Diazoxide responsive**	**Focal vs. diffuse**	**Mode of inheritance**	**Pathogenicity**
**CHI DUE TO DEFECTS IN CHANNEL AND TRANSPORTER PROTEINS**							
*ABCC8 KCNJ11*	SUR1 Kir6.2	K_ATP_ channel; regulation of channel gating	11p15.1	± No No	Diffuse Focal Diffuse	Monoallelic dominant Monoallelic recessive (Paternally inherited) Biallelic recessive	Yes
*CACNA1D*	CACNA1D	Encodes L-type voltage-gated calcium channels that play a pivotal role in the regulation of insulin secretion	3p21.1	Yes	Diffuse	Sporadic	±
*SLC16A1*	MCT1	Mediates the transport of lactate and pyruvate across cell membranes	1p13.2	±	Diffuse	Monoallelic dominant	Yes
**CHI DUE TO ABNORMALITIES IN METABOLIC PATHWAYS**							
*GLUD 1*	GDH	Central role in nitrogen metabolism, catalyses the oxidative deamination of 1-glutamate to 2-oxoglutarate	10q23.3	Yes	Diffuse	Monoallelic dominant	Yes
*GCK*	GCK	Important regulatory role in glucose metabolism	7p15-p13	±	Diffuse	Monoallelic dominant	Yes
*HADH*	HADH	Catalyzes the reversible dehydrogenation of 3-hydroxyacyl-CoAs	4q22-q26	Yes	Diffuse	Biallelic recessive	Yes
*UCP2*	UCP2	Control of pathway involved in dissipation of the proton electrochemical gradient across the inner mitochondrial membrane	11q13.4	Yes	Diffuse	Monoallelic dominant	±
*HK1*	HKI	Catalyzes the first step in glucose metabolism, using ATP for the phosphorylation of glucose to glucose-6-phosphate	10q22.1	Yes	Diffuse	Monoallelic (somatic) dominant	Yes
*PGM1*	PGM1	Catalyzes the transfer of phosphate between the 1 and 6 positions of glucose	1p31.3	No	Diffuse	Biallelic recessive	Yes
*PMM2*	PMM2	Encodes phosphomannomutase, an enzyme essential for the synthesis of GDP-mannose	16p13.2	Yes	Diffuse	Biallelic recessive	Yes
**CHI DUE TO DEFECTS IN TRANSCRIPTION FACTORS**							
*HNF4A*	HNF4α	Regulates genes largely involved in the hepatic gluconeogenic program and lipid metabolism	20q13.12	Yes	Diffuse	Monoallelic dominant	Yes
*HNF1A*	HNF1α	Binds to a sequence required for hepatocyte-specific transcription of the genes for the alpha and beta chains of fibrinogen and alpha-1-antitrypsin	12q24.31	Yes	Diffuse	Monoallelic dominant	Yes
*FOXA2*	HNF 3β	Transcription factor required for notochord formation during embryonic development involved in endoderm-derived organ system	20p11.21	Yes	Diffuse	Sporadic	Yes

*SUR1, sulfonylurea receptor 1; Kir6.2, inwardly rectifying potassium channel 6.2; GDH, glutamate dehydrogenase; GCK, glucokinase; HADH, 3-hydroxyacyl-CoA dehydrogenase; MCT1, monocarboxylate transporter 1; UCP2, uncoupling protein 2; HNF1A, hepatocyte nuclear factor 1A; HNF3β, hepatic nuclear factor 3β; HNF4A, hepatocyte nuclear factor 4A; HK1, hexokinase 1; PGM1, phosphoglucomutase 1; PMM2, phosphomannomutase 2; CACNA1D, Calcium voltage-gated channel subunit α1 D; FOXA2, forkhead box protein A2*.

**Figure 1:**
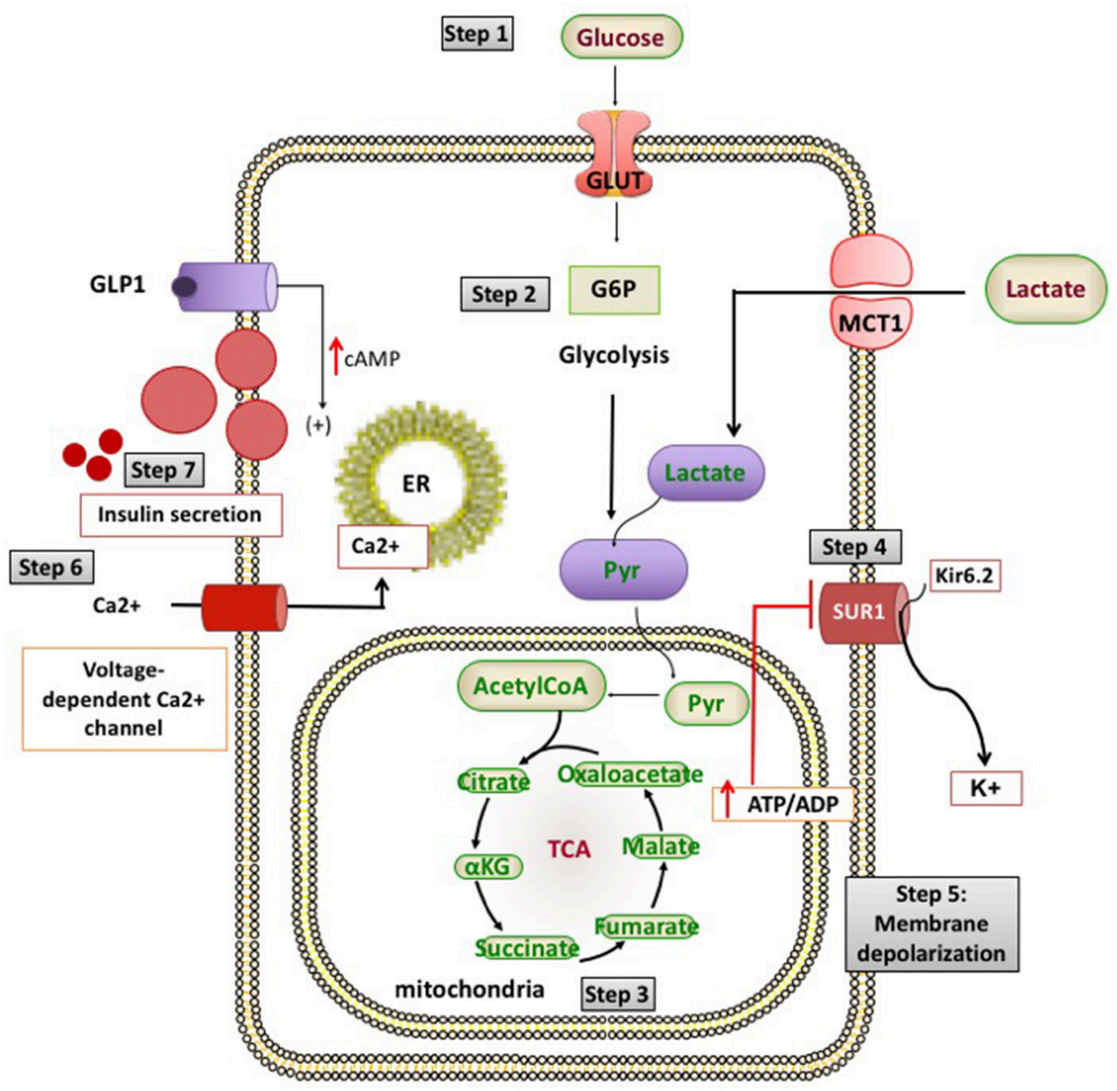
Channel and transporter proteins involved in the process of insulin secretion from pancreatic β-cells. ADP, adenosine diphosphate; ATP, adenosine triphosphate; Ca^2+^, calcium ions; cAMP, cyclic adenosine monophosphate; G6P, glucose 6-phosphate; GLP1, glucagon like peptide 1; GLUT 2, glucose transporter 2; K^+^, potassium; Kir6.2, inward rectifier potassium channel 6.2; MCT1, monocarboxylate transporter 1; Pyr, pyruvate; SUR1, sulfonylurea receptor 1; TCA, tricarboxylic acid.

**Figure 2:**
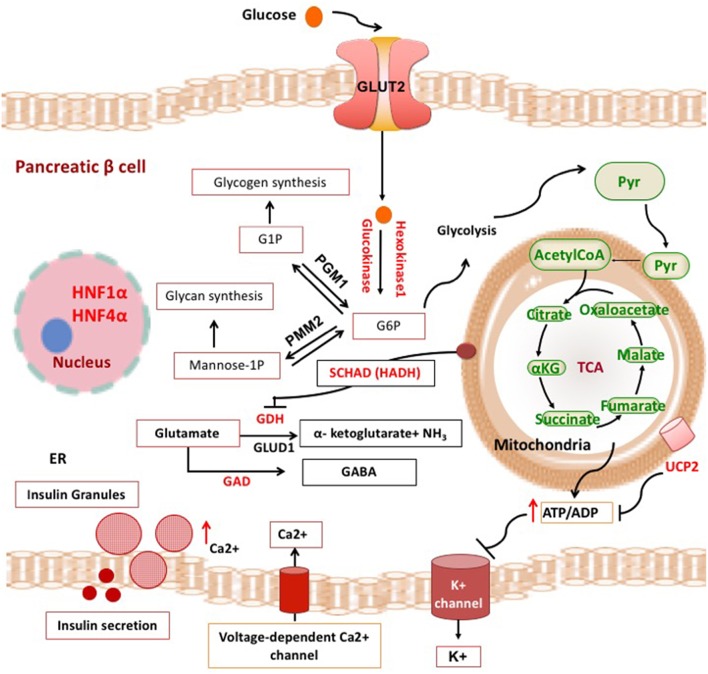
Metabolic pathways and transcription factors related to the development of CHI. ADP, adenosine diphosphate; ATP, adenosine triphosphate; αKG, alpha ketoglutarate; Ca^2+^, calcium ions; ER, endoplasmic reticulum; G1P, gluscose-1-phosphate; G6P, glucose 6-phosphate; GABA, γ-aminobutyric acid; GAD, glutamate decarboxylase enzyme; GDH, glutamate dehydrogenase; GLUT, glucose transporter; HADH, hydroxyacyl-CoA dehydrogenase; HNF1α, hepatocyte nuclear factor 1α; HNF4α, hepatocyte nuclear factor 4α; K^+^, potassium; NH3, ammonia; PGM1, phosphoglucomutase 1; PMM2, phosphomannomutase 2; Pyr, pyruvate; TCA, tricarboxylic acid; UCP2, mitochondrial uncoupling protein 2.

Broadly, these genetic defects leading to unregulated insulin secretion can be grouped into four main categories. The first group consists of defects in genes encoding the pancreatic K_ATP_ channels (*ABCC8* and *KCNJ11*) and other channel/transporter proteins (*KCNQ1, CACNA1D, SLC16A1*) (Figure 1). Pancreatic K_ATP_ channels have a critical role in the regulation of insulin release and defects in their encoding genes cause the most prevalent and severe forms of CHI (4). The second and third categories of conditions are enzymatic gene defects (*GLUD1, GCK, HADH, UCP2, HK1, PMM2, PGM1*) and defects in genes encoding the transcription factors (*HNF1A, HNF4A, FOXA2*) leading to changes in nutrient flux into metabolic pathways which converge on insulin secretion (5) (Figure 2). The β-cell insulin release is coupled to the metabolic signals and so any perturbation in these pathways will ultimately result in inappropriate insulin secretion. Lastly, there are a large number of syndromic conditions (like Beckwith-Weidemann syndrome) which feature HH as a part of the syndrome while the underlying molecular mechanism leading to unregulated insulin secretion has yet to be clarified in most of the syndromes (6).

In this article, we review the state of the art knowledge on the genetic and molecular basis of all the different types of CHI and discuss a possible new classification of the molecular basis of CHI.

### β-Cell Glucose Sensing and the Role of K_ATP_ Channels in Insulin Release

The rise of plasma glucose levels after feeding triggers insulin release from the β-cells. The glucose enters the β-cells via the facilitated diffusion driven by the glucose transporter 2 (GLUT2), where it undergoes glycolytic phosphorylation mediated by the enzyme glucokinase (GCK, a hexokinase) and converts to glucose-6-phosphate (G6P). Recently, it has been shown that other hexokinases, particularly HK1, are also expressed in the neonatal β-cells but the gene expression is downregulated after birth during the maturation of the β-cells (7, 8).

GCK is a glucose sensor linking the extracellular concentration of glucose and its metabolism in the β-cells (9, 10). The rise of the plasma glucose leads to an enhanced GCK activity producing energy rich compounds (ATP) and results in an increase in the cytoplasmic ATP:ADP ratio. The β-cell insulin release is mostly regulated by the ATP-sensitive potassium (K_ATP_) channel located in the membrane of the pancreatic β-cells (11). These channels have a crucial role in the glucose homeostasis as they link its metabolism to the membrane electrical excitability with a consequent β-cells secretion of insulin (12, 13).

The β-cell K_ATP_ channel is a large hetero-octamer with a 4:4 stoichiometry, composed of 4 pore-forming inwardly-rectifying K^+^ channel subunits (Kir6.2) and 4 high-affinity regulatory SUR1 subunits. The Kir6.2 subunit, a “weak” inward rectifier (14), contains two putative transmembrane domains (TMD1 and TMD2), bound by an extracellular loop (H5), and cytosolic -NH2 and -COOH terminal domains containing ~70 and ~220 amino acid residues, respectively.

The Kir6.2 subunit of K_ATP_ channel affects the biophysical characteristics of the channel complex such as K^+^ selectivity, rectification, activation by acyl-CoAs and inhibition by ATP (11, 15). The SUR1 subunit provides the channels with a susceptibility to the stimulatory effects of MgADP and confers on the channels a responsiveness to pharmacological activators (diazoxide) and inhibitiors (sulfonylureas) (16). It promotes ATP hydrolysis without directly transporting the substrates and regulates the Kir6.2 activity in the K_ATP_ channel complex.

Only fully-assembled channel complexes which are properly transported to the surface of the cell membrane (trafficking) can operate correctly (17). Hence, only octameric K_ATP_ channels can be expressed on the plasma membrane surface due to the masking of the endoplasmic reticulum (ER) retention signals (RKR) exposed in partially-assembled channels (18).

The key regulators of K_ATP_ channels are the changing concentrations of intracellular nucleotides. Adenine nucleotides are capable of stimulatory interacting with Mg^2+^. However, the non-hydrolytic ATP binding takes advantage over K_ATP_ channels in the lack of Mg^2+^. The increased cytosolic ATP:ADP ratio inhibits the K_ATP_ channel activity and reduces the potassium ions efflux across the plasma membrane adjusting its potential. Thus, the basal activity of the channels produce a slow depolarization until a threshold membrane potential is reached that increases the open probability of calcium channels. This process changes the physiological conformation of the potassium channels, which results in their closure, followed by chronic plasma membrane depolarization and Ca^2+^ influx due to the voltage-gated calcium channels activation (19). The increased intracellular Ca^2+^ activates specific pathways, such as protein kinases A and C, leading to impaired glucose sensing, insulin release from the storage granules and its subsequent secretion into the bloodstream (20, 21). In a similar way, when glucose concentrations are low, the K_ATP_ channels open, causing membrane hyperpolarization and subsequently inhibited release of insulin (22).

## CHI Due to Defects in Channel and Transporter Proteins

### Pancreatic β-Cell K_ATP_ Channel Defects (*ABCC8* and *KCNJ11* Gene Mutations) and CHI

Mutations in *ABCC8* and *KCNJ11* genes account for the main genetic causes of CHI, characterized by defective pancreatic β-cell K_ATP_ channel subunits (SUR1 and Kir6.2, respectively). Both genes are mapped on the same chromosome (11p15.1), being divided by a small part of DNA (4.5 kb) (16, 20, 23). The *ABCC8* gene is a large gene spanning more than 100 kb of DNA, divided in 39 exons (20). The *KCNJ11* gene consists only 1 exon encoding a protein (Kir6.2) with a molecular weight of about 43kDa (17).

*ABCC8* or *KCNJ11* gene mutations have been found in ~50% of CHI patients (24). Based on the mutation data from the Human Gene Mutation Database (HGMD) (www.hgmd.org, accessed February 2018), 448 homozygous, compound heterozygous and heterozygous inactivating *ABCC8* mutations (25–28) and around 66 *KCNJ11* (26, 29) mutations have been reported, associated with differentially expressed clinical symptoms.

Recessive and dominant *ABCC8/KCNJ11* mutations may influence the proper function of the K_ATP_ channels either by disrupting their cell surface expression or by affecting the MgADP stimulation of their activity. As a result, in both cases, the pancreatic β-cell membrane will be continuously depolarized with uncontrolled release of insulin despite severe hypoglycaemia (15, 30). The first type of *ABCC8*/*KCNJ11* mutations affecting the channel membrane expression impairs the SUR 1 synthesis or maturation, leading to proteins which do not reach the plasma membrane. These mutations can also cause impaired SUR1 trafficking. Since the Kir6.2 expression depends on the surface co-expression of SUR1, in these cases the Kir6.2 surface expression is also disturbed and it is missing from the membrane (4). The second type of mutations produce non-functional K_ATP_ channel complexes which are insensitive to the changing MgADP concentrations and stay closed even when the glucose level drops too low (4).

#### Recessive Inactivating Mutations (Homozygous or Compound Heterozygous)

In *ABCC8* and *KCNJ11* genes generally cause the most severe cases of CHI, which usually do not respond to diazoxide therapy and often require a resection of the pancreas (31). However, some compound heterozygous mutations may cause milder hyperinsulinism which is responsive to diazoxide (32). The molecular basis of these recessive mutations comprises of defects in the biogenesis and turnover of K_ATP_ channels (33), their abnormal exit from the ER and trafficking to the cytoplasmic membrane (34), and channel changes as a result of nucleotide regulation and frequency of open-state (35).

#### Recessive *ABCC8/KCNJ11* Mutations With Defects in Channel Biogenesis and Turnover

A pulse labeling study has shown that both channel subunits have long-lived species with a half-life of about 24 h, which is in accordance with the idea for a slow assembling of the K_ATP_ channel (33). The Kir6.2 turnover is biphasic when the subunit is expressed individually, as about 60% having a half-life of 36 min, while the rest changes to species with a half-life of 26 h. Expressed alone SUR1 is long-lived in the ER with a half-life of more than 25 h. Being co-expressed, both channel subunits associate rapidly with an elimination of the rapid degradation of Kir6.2 (33). After the termination of their assembly, the channels pass to the Golgi apparatus and SURs change into the mature, completely glycosylated form.

For example, the F1388del and W91R mutations in *ABCC8* and *KCNJ11* genes, respectively, which are related to the development of a critical form of CHI in patients, change the subunit turnover rate (33). The mutant subunits connect with their relevant subunits but then dissociate and undergo a rapid degradation, affecting the K_ATP_ channel biogenesis and turnover. Homozygous patients have no functional K_ATP_ channels on the pancreatic β-cell surface, while heterozygous carriers possess a reduced complement of normal channels.

#### Recessive *ABCC8/KCNJ11* Mutations With Defects in Channel Trafficking

Channel trafficking requires the RKR signals, present in both channel subunits, to be masked during their assembly. *ABCC8* mutations (F1388del, L1544P) result in a defective trafficking as they influence the exit of the subunits from the ER (36, 37). Several *KCNJ11* mutations (Y12X, H259R, E282K) have also been described as causing a trafficking defect and truncated non-functional proteins (33, 38, 39).

#### Recessive *ABCC8/KCNJ11* Mutations With Defects in Channel Regulation

Since the SUR1 protein operates as a conductance regulator of Kir6.2, both channel subunits are susceptible to alterations in adenosine and guanosine nucleotides. The K_ATP_ channel regulation involves interactions of nucleotides at the subunits with an ATP-provoked Kir6.2 inhibition, which is opposed by the ADP-activation at SUR1. Some mutations in *ABCC8* (T1139M, R1215Q) have been reported to affect the channel conductance and lead to a loss of ADP-dependent gating, causing the ATP-induced channel inhibition (40). The R1420C mutation in *ABCC8* gene, located in NBD2 of SUR1 subunit, also reduces the channel affinity for ATP and ADP (41).

#### Dominant Inactivating *ABCC8/KCNJ11* Mutations

Show normal K_ATP_ channel assembly and trafficking to the plasma membrane (42). They are uncommon and typically lead to a milder form of CHI presented at a later age, although the phenotype may vary from asymptomatic to persistent HH (43).

Interestingly, in adult carriers dominant mutations can cause dominantly inherited diabetes (44, 45). The possible mechanisms for that have been imputed to the apoptosis of the β-cell (46) mediated by the increased cell depolarization and subsequent Ca^2+^ flow into the β-cell. However, the predisposition to diabetes later in life related to dominant mutations is controversial. Huopio et al. (45) have reported a development of impaired glucose tolerance in 75% of the mothers during pregnancy while Pinney et al. (42) have not seen such a positive trend toward diabetes in mutation carriers. More recently, we reported a family carrying a novel missense c.511C>T (p.L171F) variant in exon 4 of *ABCC8* which lead to adult onset diabetes in heterozygous carriers, while a homozygous carrier developed HH at neonatal period and diabetes later in life (47).

An important feature of the dominant inactivating *ABCC8*/*KCNJ11* mutations is the responsiveness of the patients to diazoxide treatment compared to those with a recessive condition (42, 44, 45, 48). However, dominant forms unresponsive to medications have also been described (49). For example, MacMullen et al. have reported patients with medically unresponsive diffuse CHI caused by dominant *ABCC8* mutations, where 15 of all 17 patients have need a near-total pancreatectomy (50). These patients are born large for their gestational age having a similar age at clinical presentation to that of individuals with recessive mutations. The functional analyses have shown severely impaired channel responses to diazoxide and MgADP-activation; however, the channels have normal trafficking to the membrane like mutations which result in a dominant disease responsive to diazoxide therapy (50). It has been hypothesized that the mutations change the nucleotide hydrolysis rates affecting the diazoxide activity or they alter the coupling of MgATP-hydrolysis to the activation of the channel. It is believed that the MgATP-hydrolysis lead to desensitization to the ATP inhibition, so diazoxide and MgADP stabilize the desensitized condition of the K_ATP_ channel (51).

Most of the reported dominant medically-unresponsive variants have been located in the NBDs of the SUR1 subunit, including the same gene regions as many of the dominant medically-responsive SUR1 mutations (50) as well as the regions of many of the known recessive mutations (49). It has been shown that the involvement of Walker A and B motifs of NBD1 seems to be restricted to dominant mutations unresponsive to diazoxide treatment, whereas, the mutations affecting NBD2 are associated with both diazoxide-responsive and unresponsive hyperinsulinism (51).

A dominant Kir6.2 missense mutation (F55L) causing CHI has been reported to decrease K_ATP_ channels open probability without having an influence on the channel expression (35). The reduced channel activity is related to its low response to membrane phosphoinositides/long-chain acyl-CoAs (41). Recently, a novel phenomenon has been described in a transient CHI patient, reporting the co-existance of heterozygous *ABCC8* and *KCNJ11* gene mutations (52).

The detection of a *paternally inherited monoallelic K*_*ATP*_ *channel mutation* with a post-zygotic loss of the corresponding maternal region on chromosome 11 usually cause focal adenomatous hyperplasia accounting for 30–40% of the cases with CHI (15, 25, 53, 54). The loss of heterozygosity leading to paternal isodisomy makes the β-cells channel defects biallelic in the abnormal foci, changing the channels and causing abnormal secretion of insulin into the lesion (53). The subsequent disproportion in the expression of several imprinted genes involved in cell proliferation within the 11p15 region (decreased expression of the maternal tumor suppressor genes *CDKN1C* and *H19* and expression of the paternal *IGF2*) results in focal hyperplasia of the islet cells (55, 56).

**Figure 3:**
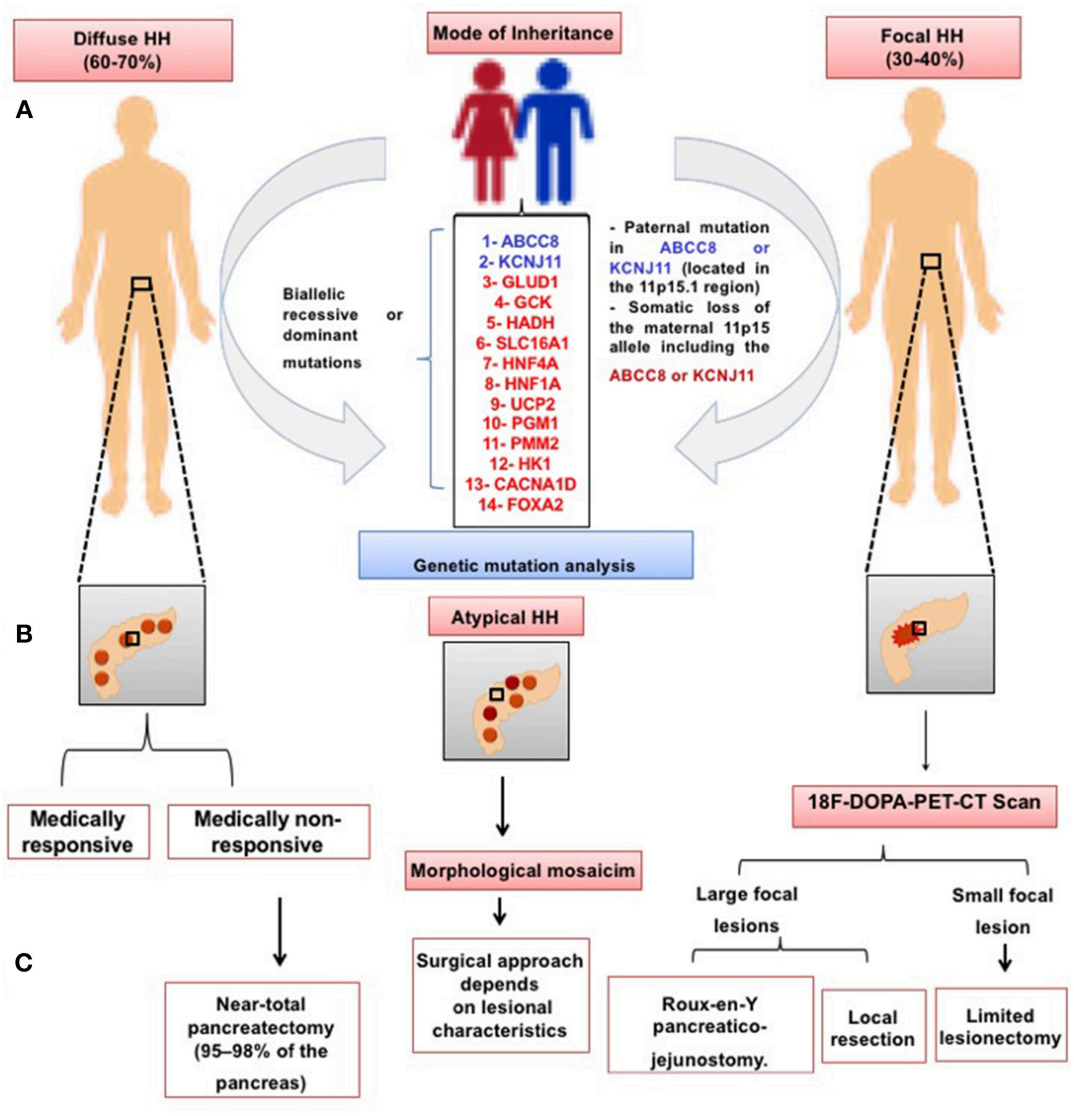
Schematic presentation of the histological subtypes of CHI. In the diffuse CHI, there is a global hyperchromatic β-cell enlargement and hyperplasia. In the focal subtype, β-cell hyperplasia is limited to a certain region of the pancreas with a superficial or deep localization. **(A)** Mode of inheritance and the genetic causes of CHI in the diffuse and focal subtype, respectively; **(B)** Schematic illustration of diffuse, atypical and focal subtypes; **(C)** Management approaches to different forms of CHI.

Focal CHI due to paternally inherited monoallelic *ABCC8/KCNJ11* mutations is usually unresponsive to medical treatment (57), although a diazoxide-responsive focal form has recently been reported (58). In the medically unresponsive children, genetic testing should be followed by 18F-DOPA PET/CT scanning in order to determine the focal or diffuse subtypes of hyperinsulinism prior to initiating the appropriate treatment to cure the patient—targeted lesionectomy in focal forms or near-total pancreatectomy in diffuse CHI (59). Figure 3 outlines the genetic causes and treatment approaches to different histological subtypes of CHI.

There are studies showing that some individuals with paternally inherited K_ATP_ mutations may respond to diazoxide or resolve spontaneously (57, 60). They are likely to have dominant acting CHI, exerting the effect of the heterozygous *ABCC8/KCNJ11* mutations by a dominant-negative mechanism leading to diffuse CHI (27, 36, 60, 61). Possible mechanisms of how heterozygous mutations can cause a diffuse disease could be a post-zygotic mutation affecting the other allele (“second hit” in the pancreas) or inability to identify a channel mutation inherited from the mother due to localization in a non-coding region of the genes (62).

A novel genetic mechanism of some atypical diffuse forms of CHI has been described in an individual with a nonsense heterozygous *ABCC8* mutation (Q54X) due to mosaic segmental paternal isodisomy (63). The absence of focal disease in this case has been explicated by the absence of uniparental disomy at the 11p15.5 region.

### Mutations in *KCNQ1* and HH

It has been reported that the pore-forming region of the α-subunit of a voltage-gated potassium channel (Kv7.1) is encoded by the *KCNQ1* gene. The channel, located in the heart, inner ear, stomach, colon and pancreatic β-cells, is crucial for ion homeostasis in the tissues. The gene mutations are associated with cardiac arrhythmias (i.e., the hereditary long QT syndrome (LQTS), deafness and defects of the gastrointestinal system (64). Recently, it has been reported the presence of HH in individuals with LQTS caused by *KCNQ1* mutations (65). These patients experience increased release of insulin during an oral glucose tolerance test with hypoglycaemic episodes following the prolonged test. Although the Kv7.1 role in the glucose metabolism is not fully ellucidated, data suggest that this channel may regulate insulin secretion by controlling the process of repolarization of plasma membrane.

### Mutations in *CACNA1D* and HH

Calcium Voltage-Gated Channel Subunit Alpha1 D (*CACNA1D)* encodes an L-type voltage-sensitive Ca^2+^ channel that affects the regulation of insulin release from the β-cells (66, 67). Activating germline mutations in *CACNA1D* gene have previously been found in patients with primary hyperaldosteronism, neuromuscular abnormalities, and transient diazoxide-responsive hypoglycaemia (68). In a recent study the same *CACNA1D* gene mutation has been shown in a patient with HH, cardiac defects and severe hypotonia (69). This mutation causes an activation of the L-type Ca^2+^ channel and leading the channel remain open at a lower membrane potential, thereby results in dysregulated insulin secretion (67, 68). Taking into consideration the role of these channels in the process of β-cell insulin release, the heterozygous c.1208G>A (p.G403D) mutation detected in present and previously published cases, suggested *CACNA1D* as one of the candidate genes for the underlying molecular genetic etiology of CHI. In addition, although the reported patient was diazoxide-responsive, considering the insulin secretion physiology from the β-cells and the role of Ca^2+^ influx from the voltage gated Ca^2+^ channels, it was suggested that calcium channel blockers would be a more effective option for the medical management of these patients (69). However, the underlying molecular mechanism leading to CHI is still not clear and requires further investigations and more experiences on HH patients with mutations of this gene.

### Mutations in *SLC16A1* Gene and CHI

The monocarboxylate transporter 1 (MCT1) catalyses the rapid transport of monocarboxylates (pyruvate and lactate) into the cells. It is encoded by the *SLC16A1* gene mapped on chromosome 1p13.2-p12. This gene consists of 5 exons and 4 introns, spanning approximately 44 kb (70). Under normal physiological conditions, there is a low MCT1 expression in the pancreatic β-cells, with minimized intracellular concentrations of pyruvate and lactate which do not increase the insulin release (67). However, dominant activating mutations in the *SLC16A1* gene promoter cause an enhanced MCT1 expression in the pancreatic islets, followed by an increased uptake of pyruvate and its metabolism in the Krebs cycle, with a subsequently increased production of ATP and insulin exocytosis (71).

The activating *SLC16A1* mutations cause CHI known as exercise-induced hyperinsulinism. The condition is characterized by hypoglycaemic episodes which usually occur within 30–45 min. after strenuous activity, in response to the accumulation of pyruvate and lactate, acting as insulin secretagogues. Sometimes diazoxide therapy may not prevent hypoglycaemia, so avoiding intense physical activity and carbohydrate intake during or after the exercise may require as a part of the treatment (72).

Although previously reported mutations have been limited to the *SLC16A1* promoter region, recently the first intragenic heterozygous mutation (c.556C>G, p.L186V) has been described in a child with CHI (73). This mutation has been found to be “probably damaging” leading to gene overexpression or baseline low expression of a mutant transport protein, followed by its enhanced transport into the pancreatic cells with subsequent increase in insulin release (73).

## CHI Due to Abnormalities in Metabolic Pathways

Other known genetic causes of CHI involved in β-cell dysregulation and abnormal insulin secretion have been identified. These involve defects in metabolic pathways converging on insulin secretory mechanisms.

### Mutations in *GLUD1* Gene and CHI (Hyperinsulinism/Hyperammonaemia Syndrome)

The *GLUD1* gene is mapped on chromosome 10q23.3. The gene consists of 13 exons and encodes the homohexameric enzyme glutamate dehydrogenase (GDH) (74), present in the mitochondrial matrix and mainly expressed in the liver, kidneys, brain and pancreas (75). GDH catalyses the reversible oxidation of the amino acid L-glutamate to α-ketoglutarate (αKG) and ammonia in the liver and kidney requiring NAD^+^ or NADP^+^ as a co-enzyme. In the pancreatic β-cell GDH catalyses the synthesis of αKG, a substrate for the citric acid cycle and causes an increased intracellular ATP:ADP ratio, activating the K_ATP_ channel with subsequent cell depolarization and insulin exocytosis (76, 77). GDH activity can be influenced by allosteric effectors working as activators (ADP and leucine) or inhibitors (GTP and ATP) (78).

Activating “*de novo*” or dominant missense *GLUD1* mutations decrease the enzyme sensitivity to the GTP allosteric inhibition and upregulate its activity in the presence of amino acid leucine, followed by increased insulin secretion (76). Interestingly, in a mutant GDH mouse model carrying the H454Y mutation, in addition to the loss of GTP inhibition on GDH activity, there is also an inhibition of glucagon secretion which contributes to the hypoglycaemic phenotype (79).

Activating *GLUD1* mutations are the second most common cause of CHI. They are identified mainly in the GTP allosteric binding site (exons 11 and 12) (80). Mutations have also been found in the catalytic site (exons 6 and 7), leading to a regular enzyme activity but reduced sensitivity to GTP (81). The exon 10 in the antenna-like region is the third domain where *GLUD1* mutations have been reported (82). The mutations cause a hyperinsulinism/hyperammonaemia syndrome (HI/HA), which is a protein/leucine-sensitive form of CHI (83, 84). It is characterized by dysregulated insulin release and persistent elevated production of ammonia (mainly in the kidney) (85).

The HI/HA syndrome, a milder form of CHI, usually presents in late infancy and early childhood. It is characterized by a normal birth-size, fasting or postprandial protein/leucine-stimulated hypoglycaemia, with persistent but asymptomatic hyperammonaemia (86, 87). The latter biochemical characteristic (hyperammonaemia) is typical for the syndrome. The ammonia levels are usually 3–5 times above the normal values but the hyperammonaemia does not appear to cause symptoms of ammonia toxicity such as lethargy, headache, vomiting, coma, etc. However, some patients (mainly those with mutations in exons 6 and 7) have episodes of epilepsy, suggesting that the condition may have a direct effect on the brain (86, 88). The suspected mechanisms for the pathogenesis of generalized epilepsy, learning disorders and developmental delay in HI/HA syndrome involve the influence of the persistent hyperammonaemia, hypoglycaemic injury of the brain and shift in the glutamate metabolism leading to decreased synthesis of inhibitory neurotransmitter γ-amino-butyric acid (GABA) in the brain due to GDH hyperactivity (88). However, there is no confirmative data for the association between ammonia levels and the risk for epilepsy or other neurological sequelae in these patients. Furthermore, there are individuals with *GLUD1* mutations who have normal ammonia concentrations, probably as a result of the mosaicism for the GDH activity(86).

Recently, a study has reported the first homozygous activating mutation of *GLUD1* in a neonate who has presented with severe hypoglycaemia, hyperammonaemia, and seizures soon after birth (89). The mutation analysis has shown a novel frameshift mutation (c.37delC) inherited from the asymptomatic mother and a *de novo* activating mutation (p.S445L) near the GTP binding site, leading to a more severe loss of the GTP inhibiting effects on GDH (89).

### Mutations in Glucokinase (GCK) Gene and CHI

The *glucokinase* (*GCK*) gene is mapped on chromosome 7 and contains 12 exons encoding for a monomeric protein with a molecular weight of about 52 kDa. The gene has tissue specific promoters, responsible for the transcription initiation in a mutually exclusive manner in distinct tissues such as the pancreas, liver, and the brain (90).

The GCK is a hexokinase isoenzyme, that has a substantial role in the carbohydrate metabolism by acting as a glucosensor for the β-cells, regulating glucose-induced insulin release. It mediates the phosphorylation of glucose molecules on carbon 6 to produce G6P, which is the first step in glycolysis (91). GCK activity has a crucial role in coupling plasma glucose levels to insulin release due to its biochemical characteristics such as a low affinity for glucose, lacking inhibition by G6P and a cooperativity with respect to glucose (92).

Heterozygous *GCK* activating mutations cause an increased glucose affinity of the enzyme leading to hyperinsulinism. So far, 15 activating mutations associated with HH have been reported, the majority of which group at the allosteric activator domain of the enzyme (91, 93, 94). The latter is the site where small-molecule activators bind, converting the GCK enzyme from its closed (active) to an open inactive state, including three intermediate stages (95). Natural ligands and activating mutations delay the changes in the shape of the GCK macromolecule when passing through the intermediate stages, suggesting the pivotal role of the allosteric domain in regulating the enzyme activity (96, 97).

Patients presenting with GCK-CHI have a wide spectrum of clinical presentations with a varying severity of the symptoms. Children usually have a family history of hypoglycaemia and age at clinical diagnosis ranging from birth to adulthood (98–101). Although most of the reported *GCK* mutations lead to CHI responsive to diazoxide therapy (98, 99), some patients may need more aggressive treatment to control hypoglycaemia such as octreotide administration or surgery (100, 102). For example, the Y214C mutation in the putative allosteric activator site has been found in a patient with medically-unresponsive CHI (103).

### Mutations in HADH Gene and CHI

The oxidation of fatty acids is an essential process that generates ketone bodies serving as alternative metabolic substrates, used in the state of prolonged fasting. The enzyme L-3-hydroxyacyl-CoA-dehydrogenase (HADH) is a mitochondrial oxidoreductase that catalyses the third step of fatty acid β-oxidation, converting the 3-hydroxyacyl–CoA to 3-ketoacyl-CoA. The coding *HADH* gene is mapped on chromosome 4q22-26 and comprises 8 exons spanning approximately 49 kb (104). Its expression in the β-cells is controlled by different transcription factors, pivotal for the correct cellular differentiation and function (105).

Recessive inactivating *HADH* gene mutations have been described in CHI patients leading to a reduction in the enzyme levels (106–108). The HADH deficiency results in a loss of its inhibitory effect on GDH, causing a GDH overstimulation, followed by an increased intracellular ATP and enhanced secretion of insulin (109). This GDH activation is restricted mainly to the pancreatic islets and does not lead to elevated ammonia levels compared to the cases with HI/HA syndrome. Therefore, *HADH* mutations cause a protein/leucine-sensitive CHI due to the close link between the oxidation of fatty acids, amino acid metabolism and insulin release (110).

Patients may have heterogeneous clinical presentations which vary between mild late-onset fasting or protein/leucine-sensitive hypoglycaemia and severe hypoglycaemia soon after birth (106, 108, 111). Some affected individuals may also have abnormally raised metabolites of acylcarnitine such as plasma 3-hydroxybutyrylcarnitine and urinary 3-hydroxyglutaric acid, with no signs of liver or cardiac dysfunction (107, 110). Most children respond to diazoxide therapy, and genetic testing is suggested when patients are from consanguineous families and negative for K_ATP_ channel mutations (112).

### Mutations in the Mitochondrial UCP2 Gene and CHI

The uncoupling protein 2 (UCP2) belongs to an inner mitochondrial anion-carrier family and is encoded by the *UCP2* gene. It is widely expressed in many tissues, including the pancreas (113, 114). UCP2 facilitates the transfer of protons across the inner membrane of mitochondria, thereby separates the mitochondrial oxidative phosphorylation from the synthesis of ATP. Since the increased intracellular ATP synthesis is the key stimulator for sensing glucose and insulin release from the pancreatic islets, the expression of UCP2 decreases the ATP synthesis and negatively regulates the glucose-induced release of insulin (114–116). Furthermore, it has been reported that the UCP2 overexpression in isolated rat β-cells downregulates the ATP synthesis and inhibits the glucose-mediatied secretion of insulin, while in UCP2 knockdown models glucose mediated insulin secretion has shown to be enhanced (117–119). Therefore, inactivating heterozygous mutations of this gene would enhance the glucose oxidation and increase intracellular ATP synthesis leading to inappropriate insulin secretion (114, 118).

CHI due to *UCP2* mutations can present with a variable clinical phenotype ranging from transient CHI to prolonged diazoxide-responsive HH (114, 120, 121). In one study *UCP2* variants were found in 2.4% of 211 diazoxide-responsive CHI patients (121). However, in a more recent study, no protein truncated variants have been detected in the *UCP2* gene among 206 diazoxide-responsive CHI patients (122). The only variant detected was considered to be a common polymorphism suggesting the necessity of further investigations of the *UCP2* role in CHI.

### Somatic Overexpression of Hexokinase 1 (HK1) and CHI

Hexokinases are a group of enzymes that catalyse the initial step in the glucose utilization, phosphorylating the hexoses (including glucose) due to the enzyme ability to transfer a phosphate group from ATP to the substrates. The phosphorylated glucose (G6P) is the most important product which serves as an intermediate substrate for glycolysis, glycogen synthesis and pentose phosphate pathway. There are four hexokinase isoenzymes (HKI-IV) encoded by different genes, which are expressed at a different rate in various tissues (123). Of those, the HK-IV or GCK, has a much lower affinity for glucose and it is expressed only when an excessive amount of glucose is present. However, HK-I has about 50-fold higher affinity for glucose and at the low glucose levels, HK-I is the dominant isoenzyme catalyzing the glucose phosphorylation. The *HK1* gene, mapped on chromosome 10q22.1, encodes the enzyme HK-I. This enzyme predominatly serves G6P to the glycolytic pathway for energy production. Its enzymatic activity is allosterically inhibited by the end-product, G6P. The elevated G6P plays a signal transducing role indicating the lack of requirement of G6P for energy production, thus suppressing the expression of *HK1*. On the other hand, the *HK1* expression at low glucose levels is silenced in the pancreatic β-cells preventing from inappropriate insulin release (8).

Recently, in a large family with dominant CHI, the responsible region has been mapped on chromosome 10, containing 48 genes (124). The first cases from this family had been reported by McQuarrie in 1954 as “idiopathic infantile hypoglycaemia” (120). Pinney et al. have investigated this large four-generation Northern European family with multiplex members with diazoxide-responsive hypoglycaemia and detected three novel variants in the *HK1* non-coding regions, suggesting the possibility of an inhibition of the *HK1* suppression in the β-cells as a result of a mutation (124). In an *in-vitro* study evaluating the pancreatic specimens of five CHI patients the role of the *HK1* overexpression has been shown in a group of β-cells (125). In these patients, although the K_ATP_ channels were functional, there was an inappropriate insulin release in the presence of hypoglycaemia (125).

### Mutations in Phosphoglucomutase 1 (PGM1) Gene and CHI

Phosphoglucomutases (PGM) are a group of enzymes that catalyse the interconversion of G6P and G1P (126). There are five different isoenzymes of PGM encoded by different genes. The enzyme phosphoglucomutase-1 is encoded by the *PGM1* gene, mapped on chromosome 1p31. It is a phosphotransferase that catalyses the reversible phosphate transfer between positions 1- and 6- of the glucose molecule, thus being involved in the glycogen synthesis, glycogen degradation and glycoprotein synthesis (126). Mutations in *PGM1* typically show a broad range of clinical manifestations resembling glycogenosis (Type XIV) as well as mixed-type congenital disorders of glycosylation (CDG type1t) (127–130). Recently, Tegtmeyer et al. have shown 19 patients from 16 families with a variety of clinical features suggesting CDG and atypical isoelectric focusing findings (128). Interestingly, the first clinical finding at birth (in 16 of 19 patients) was a bifid uvvula, but later the patients developed various manifestations including malignant hyperthermia, hepatopathy, secondary hypogonadism, short stature, hypoglycaemia, dilated cardiomyopathy and cardiac arrest. These patients were found to have recessive inactivating *PGM1* mutations, which has previously demonstrated to be related to the development of hypoglycaemia, similar to glycogenoses (127, 128). The *PGM1* mutations normally cause fasting hyperketotic hypoglycaemia, but in some cases a postprandial hypoketotic HH has been reported (131). It is proposed that the *PGM1* mutations cause HH due to the decreased threshold for glucose-mediated insulin release from the pancreatic cells (131). A later report (132) on two of the patients from the publication by Tegtmeyer (128) has shown more pronounced hypoglycosylation in childhood than in adults with a total PGM1-mRNA level reduced to 0.25% of that of normal controls. The study has also reported that hypoglycaemia might occur in *PGM1* deficient patients by starving and would be exaggerated by strong exercise (132).

### Mutations in Phosphomannomutase 2 (PMM2) Gene and CHI

The enzyme phosphomannomutase 2 (PMM2) is involved in the N-glycosylation of proteins leading to glycopreotein synthesis. In the process of N-glycosylation large carbohydrate molecules (glycans) are covalently-attached to the N-terminal of the proteins (133). Although, the N-glycosylation does not change the conformation of protein structures, it reduces their dynamic fluctuation, leading to an enhanced protein stability (133). The PMM 2 enzyme is encoded by the *PMM2* gene, mapped on chromosome 16p13.2. The enzyme is a phosphotransferase catalyzing the interconversion of mannose-6-phosphate to mannose-1-phosphate which constitutes one of the early steps of the glycosylation process. Homozygous recessive mutations of *PMM2* are responsible for a variety of symptoms related to many organ systems, which include CDG type 1a (134, 135). The severity of the disease is closely associated with the remaining enzymatic activity, therefore, it is extremely variable ranging from a lethal embryopathy to a mild subclinical disease diagnosed in adulthood (136, 137). HH has previously been reported as a part of the wide-range manifestations of CDG type Ia (138). Recently, in 17 children from 11 unrelated families, a recessively inherited mutation (c.-167G>T) found in the *PMM2* gene promoter has been shown to result in a medically-responsive form of hyperinsulinism, associated with congenital polycystic kidney disease (139). This mutation changes the formation of the tissue-specific chromatin loop with an organ-specific gene expression which could explain the selective organ involvement in this condition. The functional analysis of the mutation has revealed dysregulated insulin secretion. Therefore, the *PMM2* gene might take a part in regulating insulin release from the pancreatic islets.

## CHI Due to Defects in Transcription Factors

### Muations in *HNF1A&4A* and CHI

Transcription factors for nuclear hormone receptors (hepatocyte nuclear factors 1α and 4α (HNF1α and HNF4α*)* are expressed in the β-cells regulating the glucose-induced secretion of insulin (140–142). These factors are encoded by the *HNF1A* and *HNF4A* genes, respectively. Heterozygous inactivating variants of the genes cause two distinct conditions—CHI in newborns and maturity onset-diabetes (MODY type 1 and 3) in later life (143–146). This type of CHI due to *HNF1A/HNF4A* gene mutations is characterized with macrosomia and a clinical severity varying form mild transient hypoglycaemia to severe HH responsive to diazoxide therapy (25, 143, 147–149). In addition, a glycogenosis-like phenotype, characterized with glycogen accumulation in red blood cells, elevated liver enzymes and increased hepatic echogenicity on ultrasound examination, has been reported in CHI due to *HNF4A* gene mutations (150, 151). Although CHI due to *HNF1A* and *HNF4A* mutations is rare, in some series of diazoxide-responsive HH, these mutations have been shown as one of the most common genetic etiology (149, 152). The exact mechanisms by which the mutations in *HNF4A* and *HNF1A* could cause CHI are yet to be elucidated.

### Mutations in FOXA2 and CHI

The forkhead box A2 transcription factor (FOXA2), also known as hepatocyte nuclear factor 3β (HNF3β), is encoded by *FOXA2* gene, localized on chromosome 20p11.21. It is required for the embryogenesis and organogenesis of endoderm-derived tissues including pancreas and forebrain structures including pitutary gland (153–157). FOXA2 is a major upstream regulator of Pdx1 which is a transcription factor implicated in the pancreas development (155, 158). In mouse models it has been demonstrated that *foxa2* is necessary for the pancreatic α-cells differentiation and tissue-specific *foxa2* ablation leads to an imbalance in pancreatic β/α-cell ratio, profound hypoglucagonemia, inappropriate hyperinsulinaemia and hypoglycaemia (105, 155, 158, 159). *FOXA2* also regulates the expression of *KCNJ11* and *ABCC8* genes and has a binding site on the intronic region of *HADH* gene playing a role in its transactivation (154, 157, 159).

The effects of *FOXA2* mutations on the pituitary function have previously been shown in cases with deletions of the proximal 20p11 region (160, 161). The first mutation of *FOXA2* causing both pituitary dysfunction and CHI was recently reported in a case with congenital hypopituitarism, HH and endoderm-derived organ abnormalities (154). In this report a “*de novo”* heterozygous c.505T>C (p.S169P) variant was detected in the *FOXA2* DNA-binding domain. The patient had a specific clinical presentation including hypoplasia of the anterior pituitary, absent neurohypophysis with interrupted pitutary stalk and hypoplasia of corpus collosum, associated with multiple pituitary hormone deficiency, persistent HH, craniofacial dysmorphism and abnormalities of organs of endodermal origin, e.g., liver, lungs and gastrointestinal tract (154). The authors have shown increased pancreatic *hFOXA2* expression in the immunohistochemical analysis of human embryos and lower transcriptional activity of hFOXA2-p.S169P variant in the functional analysis. Soon after the first report, another research group has reported a “*de novo”* heterozygous p.R257L mutation in an infant with congenital hypopituitarism, HH and atypical features including coarse face, increased distance between the orbits, low-set ears, and widely-spaced nipples (157). The functional analysis of this mutation demonstrated decreased *SHH, Gli2* and *NKX2.2* gene expression suggesting the importance of *FOXA2* mutation for the abnormal findings of the pituitary gland. Besides, the partially reduced expression of *ABCC8, KCNJ11*, and *HADH* has been suggested as an evidence for the mutation effects on the developement of HH in this case. Although, the molecular basis of the HH observed in the patients has not been fully elucidated, results from previously published mouse models and latest case reports suggest that mutation in *FOXA2* could potentially be a monogenic cause of CHI.

## Hyperinsulinaemic Hypoglycaemia due to Syndromes

HH has been described in several different syndromes (6) (Table 2). In most cases the underlying biochemical and molecular basis of the HH is unknown. Beckwith-Wiedemann syndrome (BWS), the most frequent syndromic condition associated with HH, is an overgrowth disorder characterized with macrosomia, macroglossia, neonatal hypoglycaemia, hemi-hypertrophy, omphalocele. BWS patients are also prone to the development of embryonal tumors (Wilm's tumor, hepatoblastoma, neuroblastoma, rhabdomyosarcoma). BWS is linked to genetic and/or epigenetic abnormalities that alter the expression of imprinted genes on chromosome 11p15.5 (178). HH develops in approximately 50% of patients with BWS and in most cases it is transient. However, in some cases treatment with diazoxide will be required or a near total pancreatectomy (179). Although the molecular basis of HH due to BWS is not known, patients with BWS due to mosaic paternal isodisomy for chromosome 11 have the most severe hypoglycaemia (180). In one patient with BWS due to paternal uniparental disomy for this chromosome, electrophysiological studies of β-cells received at the time of surgery showed defects in pancreatic K_**ATP**_ channels (162). HH also occurs in other genetic forms of BWS but tends to be milder and shows marked clinical heterogeneity even with the same genetic cause (181). In some BWS patients, HH may be the main clinical presentation (182).

**Table 2:** Syndromic and metabolic causes of hyperinsulinaemic hypoglycaemia.

	**Gene**	**Chromosome**	**Mode of inheritance**
**PRENATAL AND POSTNATAL OVERGROWTH SYNDROMES**			
Beckwith-Wiedemann syndrome (162)	*IGF2/H19/* *CDKN1C/* *KCNQ1OT1*	11p15.5-15.4	Autosomal dominant Sporadic Paternal uniparental disomy (patUPD)
Sotos syndrome (163)	*NSD1* *NFIX*	5q35.2-35.3 19p13.3	Autosomal dominant Sporadic
Simpson-Golabi-Behmel syndrome (164)	*GPC3*	Xq26	X-linked
Perlman syndrome (165)	*DIS3L2*	2q37	Autosomal recessive
**CHROMOSOMAL ABNORMALITY SYNDROMES**			
Turner syndrome (mosaic X loss) (166)	*KDM6A*	Xp11.2	Sporadic
Trisomy 13 (167)	*CDX2, IPF1*	Trisomy 13	Sporadic
**POSTNATAL GROWTH FAILURE SYNDROMES**			
Kabuki syndrome (168)	*KMT2D* *KDM6A*	12q13.12 Xp11.3	Autosomal recessive Sporadic
Costello syndrome (169)	*HRAS*	11p15.5	Autosomal dominant Sporadic
**CONTIGUOUS GENE DELETION AFFECTING THE ABCC8 GENE**			
Usher syndrome (170)	*USH1C*	11p15.1	Autosomal recessive
**SYNDROMES LEADING TO ABNORMALITIES IN CALCIUM HOMOEOSTASIS**			
Timothy syndrome (171)	*CACNA1C*	3p21.1	Autosomal dominant Sporadic
**INSULIN RECEPTOR MUTATION**			
Insulin resistance syndrome (Donohue syndrome) (165)	*INSR*	19p13.2	Autosomal recessive
**CONGENITAL DISORDERS OF GLYCOSYLATION (CDG)**			
CDG Type Ia (138)	*PMM2*	16p13.2-13.3	Autosomal recessive
CDG Type Ib (172)	*PMI*	15q22-24	Autosomal recessive
CDG Type Id (173)	*hALG3*	3q27	Autosomal recessive
**OTHER CAUSES**			
Congenital central hypoventilation syndrome (174)	*PHOX2B*	4p12	Autosomal dominant Sporadic
Tyrosinemia type 1(175)	*FAH*	15q25.1	Autosomal recessive
Poland syndrome (176)	*UCMA*	10p13-14	Sporadic
CHARGE syndrome (177)	*CHD7*	8q12	Autosomal dominant

Kabuki syndrome is a rare genetic multisystem disorder characterized by developmental delay, “peculiar” face with elongated palpebral fissures, eversion of the lateral part of the lower eyelids, dermatoglyphic abnormalities with persistent fingertip pads, multiple congenital skeletal and visceral anomalies. Most of the cases with Kabuki syndrome have been associated with mutations in *MLL2* and *KDM6A* genes. HH has been reported in a few patients with Kabuki syndrome (168, 183, 184). However, a group has recently identified 10 patients with the syndrome (5 with MLL2 and 5 with KDM6A mutations) associated with HH. Following description of these 10 patients the authors have analyzed 100 HH patients with unknown etiology and detected Kabuki syndrome in one of those patients (1%) (185). They, therefore, have suggested that Kabuki syndrome might account for the etiology of HH in more patients. Nevertheless, the role/s of MLL2 and KDM6A in insulin release and glucose physiology is/are not ellucidated.

Sotos syndrome is also an overgrowth disorder. More than 75% of the cases with Sotos syndrome are due to intragenic mutations and deletions of the nuclear receptor binding SET-Domain 1 (NSD1) which is located at chromosome 5q35. Transient HH has been identified in several patients with Sotos syndrome (163, 186). NSD1 is not known to be directly involved in regulating insulin secretion but patients with Sotos syndrome have alterations in the IGF-1 axis which could play a role in β-cell hyperplasia (187).

Turner syndrome (partial or total monosomy X) leads to short stature in females and is associated with impaired glucose tolerance later in life. Until recently, only three patients had been described with mild diazoxide-responsive HH (166, 188, 189), all being mosaic and having the ring X chromosome. Therefore, the abnormal mosaic expression of unknown X-chromosomal gene(s) in the β-cells was suggested leading to HH. However, recently, Gibson et al. reported that patients with Turner syndrome account for 10 out of 1,050 patients with HH suggesting a frequency of 48 times more than expected (190). The authors showed that a half of the girls with Turner syndrome and HH were medically unresponsive, with 3 patients requiring a partial or near-total pancreatectomy. Furthermore, all patients with HH had at least one monosomic cell line for the X–chromosome. In the same study the authors also demonstrated increased cytosolic calcium and enhanced amino acid-induced secretion of insulin in isolated pancreatic islets from 1 case and mouse islets exposed to a KDM6A inhibitor. They suggested that the KDM6A haploinsufficiency caused by mosaic X chromosome monosomy may explain the HH in Turner syndrome.

Timothy syndrome is a rare multi-system disorder characterized by lethal arrhythmias, congenital heart defects, syndactyly, immune deficiency, intermittent HH, intellectual disability, autism and autistic spectrum disorders. It is due to novel Ca(V)1.2 missense mutations (171). This is a calcium channel protein and theoretically could be connected with the regulation of the calcium homeostasis and secretion of insulin. One patient with another calcium channel gene (*CACNA1D*) mutation has been described with HH, hypotonia and congenital heart defects (69).

Other syndromes associated with HH include Costello (169), Trisomy 13 (167), central hypoventilation syndrome (174) and recently in CHARGE syndrome (177) (Table 2).

## Conclusion

CHI is a complex condition characterized by upregulated β-cell insulin secretion leading to HH. Mutations in the *ABCC8/KCNJ11* genes of the K_ATP_ channel result in the most severe forms of CHI. Abnormal variants in several other genes with a particular importance in the regulation of insulin release and several syndromic conditions may cause milder forms of HH. The molecular cause of CHI is determined in only half of the patients. The understanding of novel molecular mechanisms of the dysregulated release of insulin will provide novel insights into the pancreatic β-cells function.

## Author Contributions

SG: literature review, wrote the section on non KATP causes of hyperinsulinism; SA-K: literature review, created all the figures and tables; HD: literature review, wrote the section on introduction and Katp channel causes of hyperinsulinism; KH: conception, planning, writing, organizing, checking and communicating.

### Conflict of Interest Statement

The authors declare that the research was conducted in the absence of any commercial or financial relationships that could be construed as a potential conflict of interest.
